# Roles of Chitosan in Green Synthesis of Metal Nanoparticles for Biomedical Applications

**DOI:** 10.3390/nano11020273

**Published:** 2021-01-21

**Authors:** Thi Tuong Vy Phan, Duc Tri Phan, Xuan Thang Cao, Thanh-Canh Huynh, Junghwan Oh

**Affiliations:** 1 Center for Advanced Chemistry, Institute of Research and Development, Duy Tan University, 03 Quang Trung, Hai Chau, Danang 550000, Vietnam; phanttuongvy4@duytan.edu.vn; 2 Faculty of Environmental and Chemical Engineering, Duy Tan University, 03 Quang Trung, Hai Chau, Danang 550000, Vietnam; 3 Industry 4.0 Convergence Bionics Engineering, Pukyong National University, Busan 48513, Korea; phanductribkhcm@gmail.com; 4 BK21 FOUR ‘New-Senior’ Oriented Smart Health Care Education, Pukyong National University, Busan 48513, Korea; 5 Faculty of Chemical Engineering, Industrial University of Ho Chi Minh City, Ho Chi Minh City 700000, Vietnam; caoxuanthang@iuh.edu.vn; 6 Center for Construction, Mechanics and Materials, Institute of Research and Development, Duy Tan University, 03 Quang Trung, Hai Chau, Danang 550000, Vietnam; huynhthanhcanh@duytan.edu.vn; 7 Faculty of Civil Engineering, Duy Tan University, 03 Quang Trung, Hai Chau, Danang 550000, Vietnam; 8 Biomedical Engineering, Pukyong National University, Busan 48513, Korea; 9 Ohlabs Corporation, Busan 48513, Korea

**Keywords:** chitosan, biomedical applications, reducing agent, size-controlling agent, shape-directing agent

## Abstract

Chitosan (CS) is a well-known stabilizer for metal nanoparticles in biomedical engineering. However, very few studies have explored other important roles of CS including reducing, shape-directing, and size-controlling. This review aims to provide the latest and most comprehensive overview of the roles of CS in the green synthesis of metal nanoparticles for biomedical applications. To the best of our knowledge, this is the first review that highlights these potentialities of CS. At first, a brief overview of the properties and the bioactivity of CS is presented. Next, the benefits of CS for enhancing the physicochemical behaviors of metal nanoparticles are discussed in detail. The representative biomedical applications of CS-metal nanoparticles are also given. Lastly, the review outlines the perceptual vision for the future development of CS-metal nanoparticles in the biomedicine field.

## 1. Introduction

Nanotechnology is thought to be the key to modern science and affects all fields of science. Nanotechnology in medicine has gained plenty of attention due to its leading role in changing the way of diagnosis and therapy [1,2,3,4,5,6]. The development of novel therapies based on nanotechnology has been increased rapidly [7,8,9,10]. Many studies have demonstrated the benefit of metal nanoparticles in new treatment strategies [11,12,13,14,15,16]. Novel methods for metal nanoparticle synthesis have gained significant interest from many research groups. Chemical and physical methods have been usually applied to obtain the metal nanoparticles. However, in the vision of the environment and human health problem, the green methods for the preparation of metal nanoparticles have been focused on more recently [17,18,19].

The shape and size of metal nanoparticles have significant effects on their properties [20,21]. The reported methods usually use a seed-assisted process [22] or capping agents (e.g., etyltrimethylammonium bromide (CTAB), etyltrimethylammonium chloride (CTAC), trisodium citrate) [23,24] to control the morphology of metal nanocrystals. After that, the surface modification is further conducted to obtain the biocompatible nanoparticles [25,26]. In some cases, this strategy could be relatively complicated and less environmentally-friendly [27,28]. Thus, the development of an alternative green synthesis method for metal nanoparticles is particularly crucial for realistic applications. In this paper, we discuss the role of chitosan (CS), which is one of the most studied polymers in nanotechnology, in the preparation of metal nanoparticles by green chemical routes.

With attractive physicochemical and biological properties, CS has been widely used as a surface coating for metal nanoparticles [29]. CS can work as a reducing agent, a shape director, or a size-controllable agent in the synthesis of metal nanoparticles. The previous studies have shown that functionalizing the surface of metal nanoparticles by CS can offer many advantages, including improving physicochemical stability [30], a drug carrier [31], controlling drug release [32], promoting muco-adhesiveness and tissue penetration [33], encouraging cell interactions [34], and enhancing antimicrobial effects [35].

The CS-metal nanoparticles have a wide range of applications in the biomedical field. Recently, photothermal therapy (PTT), which is based on the conversion of photon energy to heat by photo-absorber, and photodynamic therapy (PDT), which is based on the production of reactive oxygen species by a light-triggered photosensitizer, have been developed for cancer treatment [36]. Biocompatible CS-metal nanoparticles are good photo-absorbers for the PTT owing to the surface plasmonic of metalcore and also good photosensitizer-carriers for PDT owing to the CS layer. Additionally, the metal nanoparticles with the CS layer could be an effective drug carrier with controlled delivery ability, as reported in References [31,32]. Furthermore, owing to the strong antibacterial of CS, they can be used as an effective antibacterial agent [37,38]. CS-metal nanoparticles are not only applied in treatment. They can also work as an external agent of a diagnosis method. Recent studies have reported that these nanoparticles can be promising candidates for photoacoustic imaging (PAI) in the diagnosis of disease.

This review has been motivated to share up-to-date information on the role of CS in the green preparation of metal nanoparticles toward biomedical applications. First, we present the excellent properties and bioactivity of CS in brief. Next, we review the presentative research works on the role of CS in the synthesis of metal nanoparticles including stabilizing, reducing, shape-directing, and size-controlling. Afterward, the applications of CS-metal nanoparticles in the field of biomedicine (i.e., drug delivery, PTT, PDT, PAI, and antibacterial therapy) are assessed. Finally, we provide the perceptual vision of the role of CS for the environment-friendly synthesis of metal nanoparticles for future development in biomedicine.

## 2. Chitosan Properties

**Figure 1:**
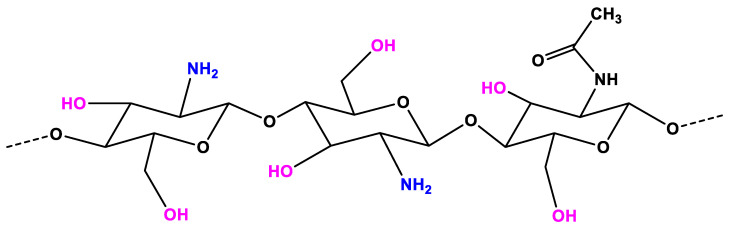
The chemical structure of chitosan (CS).

CS, which is deacetylation of chitin, is a linear polysaccharide consisting of glucosamine and N-acetylglucosamine units [39]. CS is considered as a non-toxic, odorless, biocompatible, and biodegradable biopolymer. Thus, CS is considered a renewable, sustainable, and affordable polymer [40,41]. In the structure of CS (Figure 1), an amino group together with hydroxyl groups (both principal and secondary ones) are the reactive functional groups [42]. The structure of CS and its physicochemical properties vary with the amino group through intra-molecular and inter-molecular hydrogen bonds. The molecular weight (MW, the number of sugar units over polymeric molecule) and the degree of deacetylation (DDA) are known as the main parameters that affect the CS’s properties [41]. As reported in References [43,44,45], the physicochemical characteristics, including solubility, adsorption on solids, tear strength, viscosity, elasticity, and bio-functional activities are strongly associated with the polymeric MW. The DDA affects the solubility of CS in the acid solution and the flexibility of CS molecules [46]. The CS with high DDA is more flexible and tends to shape an irregular coil with additional intramolecular hydrogen bonds inside the CS chain [41]. Consequently, the CS chain becomes less intertangled in the structure and has a more elliptical shape. The mechanical properties of the elliptical CS chain are generally less strong than those of less deacetylated microspheres in general. On the other hand, the CS chain with less deacetylation is more expanded and has stronger interactions between molecules, making the chain more intertangled. The DDA also plays an important role in the proliferation and adhesion of cells, as reported in References [47,48,49,50]. The CS with low DDA is favorable for the growth and adhesion of cells. The viscosity of the CS mixture decreases with the temperature and increases with the DDA and the concentration. The solubility of CS is dependent on the pKa and the acidic solvent’s strength. CS is soluble in a weak acidic solution, but insoluble for pH > 7.

CS is the second most abundant natural polymer and is the only natural polycation alkaline polysaccharide with a glucosamine content of more than 90% [41,51]. They are easy to be extracted from natural sources [52,53] and have a cheap price in comparison with some other polymers, such as fucoidan or hyaluronan acid. The glucosamine backbone of CS contains a high density of the amino [54], which makes CS become a very bioactive polymer. CS can be modified to enhancing the desired properties [55]. With those excellent properties, CS is a polymer that has been applied in the green preparation of metal nanoparticles for biomedicine application.

## 3. Bioactivity of Chitosan

### 3.1. Antibacterial

The antimicrobial activity of CS has been proven by many studies [35,56]. The inhibition mechanism is based on the interaction of the positive charge CS at the acidic condition with negatively charged residues of biomolecules on the surface of the bacteria cell [35]. Another possibility is that CS permeates into the cell nucleus and inhibits the RNA and protein synthesis as well as the rupture and leakage of the intracellular component [57]. Pedro et al. [58] demonstrated that electrostatic interaction of CS with polar groups dipalmitoyl phosphatidylglycerol of bacterial membranes are dominant, resulting in changes of membrane potential, elasticity, and possibly its permeability to biomolecules. In contrast, the interaction of CS and the dipalmitoyl phosphatidylcholine monolayer of mammalian cell membranes are weak and likely favored by hydrophobic interactions of the CS backbone with lipid tails. These different interaction mechanisms may explain why CS-based material are usually bactericide, but not toxic to mammalian cells.

The MW strongly affects the antibacterial properties of CS [59]. CS with low MW penetrates easily to the cell wall of bacteria. Meanwhile, the CS with high MW has a lower permeation capacity to the bacterial membrane. The antimicrobial activity of CS is also influenced by other factors such as solubility, pH, and the temperature environment.

### 3.2. Antioxidant Activity

The free radical reaction contributes to many chronic health problems [60]. Free radicals are unstable and tend to pair up with other molecules and atoms to form a stable state. Antioxidants can prevent the formation of free radicals by scavenging them, or by promoting their decomposition. Recently, the antioxidant activity of CS has been investigated by many research groups [61,62,63]. CS exhibits outstanding scavenging activity against different radical species. The mechanism of the antioxidant property of CS is based on the donating hydrogen atoms for free radicals binding [64]. Mahdy Samar et al. [65] tested an antioxidant activity of CS with various DDA and MW and observed that CS with high DDA and low MW has better antioxidant activity.

### 3.3. Anti-Inflammation

From the experimental results, many studies have confirmed that CS has anti-inflammatory and anti-proinflammatory properties. Davydova et al. [66] tested the anti-inflammatory activity of CS with high (115 kDa) and low MW (5.2 kDa). Both CS samples induced the anti-inflammatory in animal blood and suppression of colitis progress. The results showed that the anti-inflammatory activity of CS depends on structural elements. Meanwhile, the MW does not affect this activity. However, Chang et al. [67] claimed that larger MW (>29.2 kDa) CS has anti-inflammatory activity whereas smaller MW (≤29.2 kDa) CS have proinflammatory activity. Despite those previous research attempts, extensive studies still need to be carried on to understand the anti-inflammation of CS.

### 3.4. Anti-Cancer Activity

Many reports showed that CS can be a potential anti-cancer polysaccharide [68,69,70,71]. It can inhibit tumor growth by preventing tumorigenesis, inducing tumor cell apoptosis, and inhibiting tumor metastasis. Through the experimental evaluation, Park et al. [72] concluded that the MW and DDA of CS are important factors for the exhibition of anti-tumor activity in vitro. The experiments on three cancer cell lines pointed out that the CS with lower MW and higher DDA has better anti-cancer activity. Another study [73] showed that CS has a larger effect on a negatively charged tumor cell. Particularly, the decreased level of vascular endothelial growth factor receptor 2 on the tumor surface limited the growth of HepG2 cells and led to the inhibition of tumor angiogenesis.

## 4. Chitosan on the Formation and Functionalization Processes of Metal Nanoparticles

**Figure 2:**
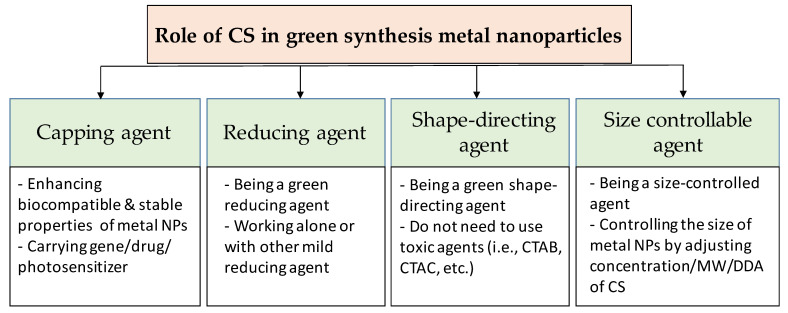
Overview of the role of chitosan in green synthesis of metal nanoparticles.

CS can affect both the formation and the functionalization processes of metal nanoparticles (Figure 2). When the cationic polymer CS is added to the reaction solution, electrostatic interaction happens between positively-charged CS with negatively-charged nanoparticles (which have the negative capping agent) [74], or the interaction occurs by absorbing CS on the metal nanoparticle surface [75], resulting in a CS shell around the nanoparticles. Regarding the formation process, the CS can add before or during the formation of the nanoparticles. Thus, CS directly affects the formation of nanoparticles. In this case, CS can act as the stabilizing agent, the reducing agent, the size-controllable agent, and the shape-direction agent for the synthesis of metal nanoparticles. For the functionalization process, CS is used to modify the surface of nanoparticles to enhance the biocompatible and carrying abilities for drugs of metal nanoparticles.

## 5. Chitosan as a Stabilizer

**Figure 3:**
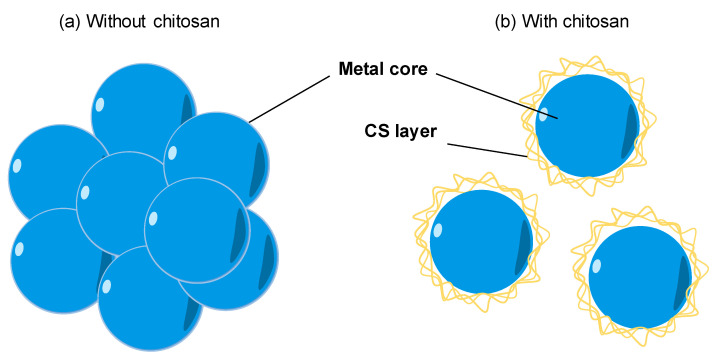
The effect of chitosan (CS) capping on the dispersion of metal nanoparticles. (**a**) The aggregation of metal nanoparticles without a CS capping agent in solution. (**b**) The good dispersion of metal nanoparticles with a CS capping agent in solution.

The natural polymers are usually chosen as a stabilizer for the synthesis of metal nanoparticles. With the availability, biocompatibility, highly positive charge, CS becomes a good stabilizer of metal nanoparticles. As shown in Figure 3a, the bare metal nanoparticles can easily aggregate in solution due to the Van der Waals interactions between raw metal surfaces. In contrast, CS is a steric barrier with a positive charge density covered around the metal. The strong electrostatic interaction among positive-charged metal nanoparticles allowed the formation of homogeneous metal nanoparticle solutions, as seen in Figure 3b. Many studies reported the good performance of CS in the role of stabilizing metal nanoparticles. For example, CS was utilized as the stabilizer to synthesis the silver nanoparticles (AgNPs) using a green approach based on an electrochemical oxidation/complexation process with UV irradiation reduction [76]. In another example, gold nanoparticles (AuNPs) were synthesized in the presence of CS and citric acid. The as-prepared AuNPs were stable in the aqueous phase without any agglomeration [30,77]. The water-soluble chitosan oligosaccharide (COS) from CS has also been widely used for coating metal nanoparticles oriented toward particular biomedical applications, including drug/gene delivery, photo-based therapy, and tissue engineering [11]. The COS was used as a green reducing agent/stabilizer for the one-pot synthesis of AuNPs for gene transfer [78]. The positive charges by the amino groups of COS improved the affinity with plasmid DNA.

## 6. Chitosan as a Green Reducing Agent

The toxic reducing agents are often used in the synthesis of nanoparticles by chemical methods, which release environmentally hazardous chemicals. The nanoparticles synthesized from chemical routes could not be directly used for biomedical applications because of the presence of toxic capping, which cannot be separated easily from the nanoparticles. To protect the environment and to be used in biomedicine, nanoparticles need to be synthesized through green methods and with green materials. Many reports have revealed that CS can act as both reducing and stabilizing agents for the green synthesis of AgNPs [79,80], copper nanoparticles (CuNPs) [81,82], and AuNPs [80,83,84]. Carapeto et al. [85] put the effort to unravel the reaction mechanism of Ag ion by CS with the help of UV/Vis absorption and x-ray photoelectron spectra analysis. The experimental results showed that the very fast Ag reduction in CS aqueous solutions happens in the early stages even at room temperature, and the reaction happens faster when the temperature reaction was increased. The oxidation of alcohol or glucosidic groups of several functional groups in CS provides the free electron to reduce Ag^+^ to Ag^0^ and to form the carbonyl groups. By the UV-Vis spectra (absorption peak at ~262 nm, which indicates π* ← n transition in a carbonyl group) and the X-ray photoelectron spectroscopy (XPS), the authors proved that the main products in the reaction medium are carbonyl groups. The coating/wrapping of the metal with the cationic CS results in the positively charged nanoparticles and long-term stability in terms of aggregation [86].

Wongpreecha et al. [87] provided another explanation for the reaction mechanism of CS. The green process was taken in an autoclave under high temperature (120 °C) and high pressure (15 psi). In a pH 4 environment (lower than pK_a_ of CS), Ag+ was coordinated with pair electrons of nitrogen and/or oxygen on the CS backbone. Then, Ag^+^ was reduced to Ag^0^ by a lone pair electron of oxygen in CS under high temperature and high pressure. CS played as a steric and electrostatic stabilizer for the resulting nanoparticles.

Creating a core-shell nanostructure is an effective strategy to enhance the performance of metal nanoparticles. The CS was also used as a green reducing agent to fabricate nanoparticles with the core-shell nanostructure for applications in the biomedicine field. Wang et al. [88] prepared a core-shell nanocomposite termed Cu@Pd-CS by the green method with natural CS. The synthesized Cu@Pd-CS showed good stability, sensitivity, and anti-interference.

## 7. Chitosan as a Size-Controllable Agent

**Figure 4:**
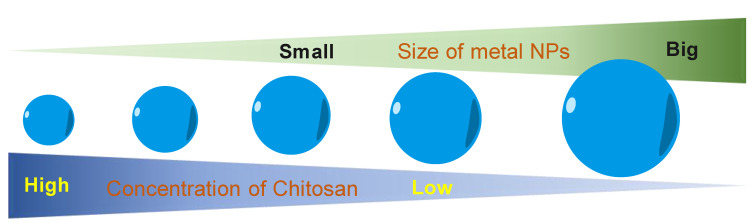
The correlation of concentration of chitosan and the size of metal nanoparticles.

Another role of CS in the green synthesis of metal nanoparticles is size control. Based on the UV–visible absorption spectrum data, Kalaivani et al. [89] observed that the AgNPs formation was efficiently increased in the presence of CS. In addition, the size of AgNPs was remarkably decreased at a higher CS concentration (Figure 4). This conclusion was again confirmed by our recent studies. The size of metal nanoparticles (i.e., PdNPs [90] and AuNS [91]) was decreasing when the added concentration of CS was increased. We proposed a hypothesis to explain how the CS affects the size of formed nanoparticles. During the formation of metal nanoparticles in the presence of CS, the positively charged CS has a strong electrostatic interaction with the metal nuclei. The higher concentration of CS leads to the stronger interaction of CS and the metal nuclei. This strong interaction inhibits the binding of precursors to the metal nuclei. Thus, metal nuclei are not able to grow more in the presence of a high concentration of CS solution.

## 8. Chitosan as a Shape-Directing Agent

In the chemical route, toxic capping agents (e.g., CTAB, CTAC, trisodium citrate) [23,24,25] are usually used as the shape-directing agent for the metal nanoparticles. Replacing them with natural products is a good strategy to enhance the biocompatibility of metal nanoparticles for sustainable biomedical applications. CS was used as a structure-directing agent for the electrodeposition of AgNPs on disposable, pencil graphite electrodes [92]. The authors observed that AgNPS has well-defined morphologies in the presence of CS. Meanwhile, AgNPs have an irregular structure in the absence of CS. By controlling the experimental conditions, various morphologies of Ag NPs such as hexahedron, leaf, and dendrites have been obtained.

In addition, the properties of CS for controlling the shape of nanoparticles can be enhanced by modifying the CS, such as by using an anionic ligand, o-carboxymethyl, or a cationic N-trimethylamine group. Different shapes of AuNPs can be formed by the controlling effect of the CS’s positive and negative charges. As a soft template, thiolate-functionalized CS can be used for the preparation of gold nano chains, nanoneedles, as well as nanoflowers, as reported in Reference [93]. The CS’s thiolgroup can extensively interact with AuNPs owned by various assemblies. Additionally, it is shown that a peptide that contains an aromatic moiety can be used to template the architecture of gold nanocrystal via self-build, as reported in Reference [94]. In another study [95], folic acid (FA) and gallic acid (GA) -N-trimethyl CS (FA-GA-TMC) was demonstrated for the self-assembly of SeNPs with a cubic shape. With the modification of CS, three important structural features can be obtained, as follows: (1) the improvement of the electrostatic interaction between the negatively charged surface of SeNPs [96] and the stabilizer’s positive charge due to the N^+^(CH_3_)_3_ group’s positive charge of the quaternized CS, (2) the creation of a π-π stacking interaction together with a rigid template through hydrophobic elements of both the GA and the FA, and (3) the last one is the hydrogen bonding groups from the GA, FA, and the CS backbone. The interaction between the hydrophilic group of N^+^(CH_3_)_3_ and the negative charge of the SeNPs’ surface creates an outward presentation of the large hydrophobic groups of the GA and FA. This allows the π-π stacking interactions and the hydrogen-bonding interactions among surrounding particles, and further open the door for the assembly into cubic-like SeNPs.

## 9. Chitosan as a Multifunctional Agent on the Preparation of Metal Nanoparticles

Recent reports provided evidence that CS can work as a multifunctional agent for the synthesis of metal nanoparticles, such as AuNPs and PdNPs. In 2019, we have developed the novel green method for the synthesis of flower-shaped porous palladium nanoparticles (PdNPs) and CS plays as multifunctional agents including a stabilizer, shape director, and size-controller in this method [90]. We set up the experiments with various amounts of CS and vitamin C, which is a green reducing agent, was used to reduce Pd^III^ to Pd^0^. In all experiments, we got the PdNPs with the flower shape even though no shape-directing agent was used. In that, CS played the important role in the formation of flower-shaped PdNPs. CS covered the surface of the PdNPs via a strong interaction with Pd nuclei after the formation under the effect of vitamin C. In the presence of CS, the Pd nuclei continuously grew in an anisotropic direction and resulted in PdNPs with a porous flower shape. The reduction process on the surface of Pd nuclei was inhibited due to the formation of the CS layer. At this time, PdNPs were completely mature. We observed that, when increasing the concentration of CS, the smaller PdNPs were obtained. A higher CS amount in the solution will lead to the quicker formation of the CS layer and the smaller PdNPs will be obtained. Based on this principle, the PdNPs with the desired size can be prepared by adjusting the amount of added CS. In the other work [91], we utilized the CS as a multifunctional agent for the green preparation of gold nano-stars (AuNSs). With highly positive charges in the low pH environment, CS binds to the core of AuNSs via strong interactions with Au nuclei. The tip has strongly grown at the loose contact points between CS and the core, facilitating the anisotropic growth of gold nanostructures and resulting in a star-like structure. In a higher pH environment, CS has a weaker interaction with Ag nuclei and are not able to control the growth of Ag nuclei. Thus, AuNSs were not successfully formed.

## 10. Applications of Chitosan-Metal Nanoparticles and Their Advantages

### 10.1. Controlled Drug Delivery Application

**Figure 5:**
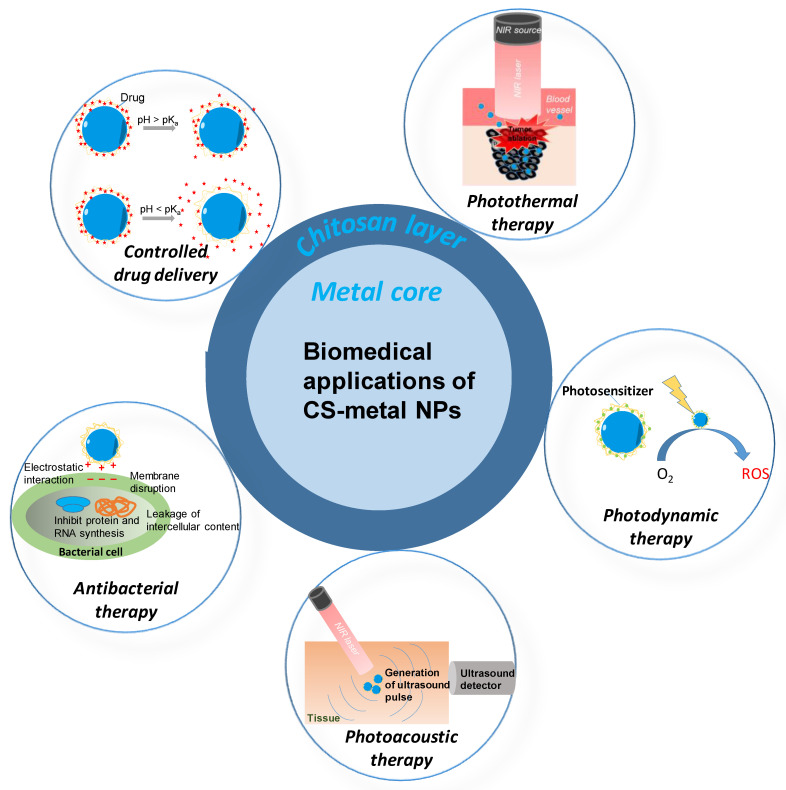
The biomedical application of CS-metal nanoparticles.

The uncontrolled drug delivery leads to the side effects of healthy tissue and the ineffective concentration of the target tissue. In the trend of the development of less toxicity and a better therapeutic drug delivery system, the formulations consisting of drugs encapsulated with CS have been widely studied [97]. The drug can be entrapped to the CS layer of CS-metal nanoparticles via chemical crosslinking, ionic crosslinking, and ionic complexation. CS with the pKa 6.5 can be able to deprotonated/protonated in a high pH /low pH environment. Thus, the CS-metal nanoparticles are a pH-responsive drug release system (Figure 5). Varukattu et al. reported the synthesis of CS coated copper oxide nanoparticles (Cs-CuO NPs) and tested their performance on loading and delivery of doxorubicin (DOX) into breast cancer cells. The 89 µg DOX was loaded to the CS matrix of CS-CuO nanoparticles [31]. The release profile showed that 35% of DOX was released at a pH of 7. In contrast, up to 90% of DOX was released at a pH of 5. A lower amount of DOX was released under the physiological pH of normal tissue and the huge amount of DOX was released at the acidic condition, which was similar to the physiological pH of cancer tissue. Thus, the pH-dependent behavior of these nanoparticles can improve the therapeutic activity of DOX-loaded nanoparticles without disturbing the normal cells.

In another example, Ma et al. fabricated Au@CS NPs by one-pot synthesis and modified them by cell membrane mimetic phosphorylcholine zwitterions polymer (Au@CS-PMPC NPs) to avoid the fast elimination of positive charge Au@CS through the monocyte phagocytic system [32]. No clear drug was leaked out of Au@CS-PMPC NPs at a pH of 9.0 for 12 h and about 28% and 33% DOX was released at a pH of 7.4 and a pH of 4.0, respectively. In the pH of 5.5 and 4.0 solution, CS was in the protonated state and the electrostatic repulsion between the positively charged CS and DOX allowed the drug releasing.

The CS-metal nanoparticles can be used as the novel carrier for insulin. Bhumkar et al. [86] synthesized the AuNPs with the reducing ability of CS. The loading efficiency of insulin on CS-AuNPs was 53%. The binding of insulin to the CS layer of CS-AuNPs plays a curial role in the release and subsequent activity. It was observed that the insulin-loaded AuNPs showed 4–5 times greater permeation as compared to free insulin. Thus, CS-AuNPs are the promising carrier for controlling postprandial hyperglycemia.

The degradability/elimination and the immunogenic response of CS-metal nanoparticles in the body have not been studied deeply yet. To transfer the drug-carrying ability of CS-metal nanoparticles to the real application, the extended studies need to be conducted.

### 10.2. Antibacterial Therapy Application

Multi-drug resistance is a big issue of modern medicine. To solve the multi-drug resistances, the scientist needs to focus on the development of novel efficient bactericidal materials. CS with the highly positive charges, CS-metal nanoparticles have great potential as cost-effective and sensitive nanosensors for Gram-negative bacteria due to the strong electrostatic interactions with lipopolysaccharides in their outer membranes. For example, CS-AuNPs was proven as effective bactericidal materials [37]. The experimental results showed the inhibition of the normal growth of highly resistant bacterial strains. The authors also provided evidence that the charge density of CS decided their antibacterial activity. This property has been associated with strong electrostatic interactions of CS with the charged surface of the lipid bilayer of bacterial cell membranes, suggesting that the action mechanism of these CS-AuNPs followed a non-specific action mechanism. The Au-CS nanocomposites, which were developed by Mendoza et al. [98], showed concentration-dependent antibacterial activities against Escherichia coli as Gram-negative and Staphylococcus aureus as a Gram-positive bacterial model. The flow cytometry and SEM studies revealed the bacterial death mechanism mediated by Au-CS colloids that may be related to cell wall disruption and intracellular content leakage. The AgNPs is well-known as an effective antibacterial nanoparticle. However, its effective concentration is quite high and has limited direct uses as an anti-bacterial agent. CS can act as a nano-carrier and also as a co-antibacterial agent toward AgNPs. Sharma et al. [38] prepared the AgNPs loaded-CS nanoparticles and tested their antibacterial performance. TEM images revealed the strong attachment of the nanocomposite to the bacteria due to their high surface area and reactivity. The dual-effects of CS and AgNPs on the bacterial membrane lead to leakage of proteins and other intracellular constituents, which resulted in the death of bacteria.

### 10.3. Photothermal Therapy Application

PTT is a photo-based therapy that has recently been developed for theranostic applications [36,99]. Their advantages include minimal invasion, fast recovery, preventing damage to normal tissues, and having very few patient complications. The CS-AuNPs were proven as an effective photothermal agent by many research groups [100,101]. We prepared the CS-coated AuNS by the green method and tested their photothermal performance on the breast cancer cells [91]. The synthesized CS-Au nano stars not only presented highly photothermal stability, high absorption in the near-infrared(NIR) region, but also exhibited excellent biocompatibility on both MDA-MB-231 and MG-63 cells. The MDA-MB-231 breast cancer cells were effectively killed by CS-Au nano stars plus the NIR laser, which proved their ability for PTT. Another study from our group reported the photothermal performance of CS-PdNPs, which were also prepared by the environmental-friendly method [90]. The MDA-MB-231 cancer cells were effectively killed by the CS-PdNPs when the photon was converted to heat, thus, demonstrating the ability of these nanoparticles for PTT.

The distribution of CS-metal nanoparticles in the tumor decide the effectiveness of PTT [102]. Thus, there is a need to develop the cancer cell targeting CS-metal nanoparticles to achieve the well-distribution of nanoparticles in the tumor. Moreover, PTT could be combined with other therapies such as chemotherapy, immunotherapy, radiotherapy, or surgery to achieve the highest results on cancer treatment [103].

### 10.4. Photodynamic Therapy Application

PDT is also a photon-based therapy with a combination of low-intensity visible light and a photosensitizer (PS). When the PS is triggered by visible light in the presence of oxygen, it produces cytotoxic agents that can kill or damage the tumor cells. With the CS layer, the CS-metal nanoparticles can carry the photosensitizer for PDT. The photosensitizer can be conjugated with a functional group of CS. Hari et al. attached Acridine orange (AO) to the AuNPs surface through glutathione to form a multi-functional nanoparticle for enhanced PDT and PTT targeting of breast cancer cells [104]. The experimental results showed two-fold increases in fluorescence intensity and faster cellular uptake and photostability than free AO. From this observation, we can realize that packing the photosensitizers in the CS-metal nanoparticle brings more effectiveness of PDT and PTT.

The visible light used in PDT cannot penetrate the tissues deeply, which restricts the application of PDT to deep cancer tissue treatment [105]. Thus, the development of photosensitizers that can absorb NIR light has been required to enhance the deepness of treatment. Besides that, the targeting delivery of PSs by CS-metal nanoparticles can also improve the effectiveness of PDT. The CS-metal nanoparticles can be conjugated with targeting agents such as antibodies, known as DNA/peptide-based linkers, to improve both target specificity and pharmacological properties [106].

### 10.5. Photoacoustic Therapy Application

PAI is future biomedical imaging with the benefit of optical resolution and acoustic penetration depth [107,108]. With its capacity to offer anatomic and functional information such as breast cancer detection, epidermal melanin measurements, brain structure, brain function imaging, blood oxygenation monitoring, and quantitative blood flow estimation, PAI has promising potentials in a wide range of preclinical and clinical applications. To enhance the signals from PAI, exogenous contrast agents are usually used. Some recent studies have demonstrated that CS-metal nanoparticles can be good exogenous contrast agents for PAI. For example, the in vitro test photoacoustic performance of CS-coated AuNS and CS-coated PdNPs, which were obtained from the green method, showed a strong photoacoustic signal [90,91]. When increasing the concentration of CS-coated metal nanoparticles, the higher photoacoustic signal was received. The results demonstrated that light-absorbing CS-metal nanoparticles could be good candidates for PAI.

## 11. Conclusions and Perspectives

CS is a kind of safe and reliable natural bioactive polysaccharide and has recently become an attractive polymeric biomaterial. The active amino and hydroxyl functional groups in the CS backbone mainly contribute to the excellent biological activities of CS. The combination of CS-organic material and metal-inorganic material can create nanoparticles with the properties of both the organic polymer and the inorganic material. These nanocomposites could bring plenty of benefits for the novel materials oriented toward biomedical applications.

CS can work as a multi-functional agent in the green synthesis of metal nanoparticles. However, there are few studies that have discovered this great potential of CS. The use of CS in the synthesis of metal nanoparticles can reduce the harmful effects on the environment and human health. This strategy is particularly important for the sustainable development of biomedical nanotechnology and other related fields. The potentialities of other natural polymers such as cellulose, fucoidan, gelatin, alginate, hyaluronan acid, etc. on the green preparation of metal nanoparticles would also be exploited. The green chemical routes would have more influence on the synthesis of nanoparticles.

As we discussed above, the properties of CS are strongly affected by the MW and DDA. Thus, to give a complete protocol for further research, the influence of MW and DDA on the formation of metal nanoparticles should be studied extensively.

With their excellent properties, we believe that the novel application of green CS-metal nanoparticles will be discovered in the near future. For example, CS-metal nanoparticles might be used for a wide range of diagnostics methods owing to the optical properties of the metalcore. CS-metal nanoparticles have a promising potential for the development of advanced imaging-guiding therapies due to the carrying ability of CS and the unique properties of a metal core. CS layer-metalcore nanostructure allows the combination of multi-modals into a single system (i.e., chemo-PTT, chemo-PDT) to enhance the effectiveness of theranostics that may be a trend of further medicine. The combination of the CS-metal nanoparticles with other natural polymers can create novel nanostructures with unique and genius properties for specific biomedical applications.

The biomedical application of CS-metal nanoparticles, which are prepared by the green method, has not been deeply investigated in comparison to the one prepared by the chemical routes. Specifically, almost all studies have stopped at in vitro studies and only a few have gone further to in vivo studies. To evaluate the effectiveness of CS-metal nanoparticles synthesized by the green method, extensive animal studies and clinical trials are suggested for future research.

## Data Availability

No new data were created or analyzed in this study.
